# A Protective Haplotype *in trans* Modifies Penetrance and Severity of *TOR1A* Dystonia

**DOI:** 10.64898/2026.09.05.26361794

**Published:** 2026-09-10

**Authors:** Roy Dayan, Orli Halstuk, Shira Yanovsky-Dagan, Jonathan Rips, Chaggai Rosenbluh, Ilana Livyatan, Michelle Grunin, Morasha Plesser-Duvdevani, Hagai Bergman, Tamir Ben Hur, James R Lupski, Deborah Raymond, Susan Bressman, Rachel Saunders-Pullman, Aloysius Domingo, Laurie J Ozelius, Shai Carmi, Tamar Harel, David Arkadir

## Abstract

**Introduction:**

Incomplete penetrance and variable expressivity characterize autosomal dominant neurological disorders, but their genetic determinants remain largely unknown. DYT- *TOR1A* dystonia provides a model for investigating incomplete penetrance. We aimed to identify modifiers of disease penetrance and severity beyond the known *TOR1A* c.646G>C modifier.

**Methods:**

In a prospective genotype–phenotype association study, we phenotyped Ashkenazi Jewish carriers of *TOR1A* c.907_909del using a prespecified five-level severity score. The primary analysis compared participants with no or minimal dystonia (scores 0–1) and severe dystonia (scores 3–4), after excluding c.646G>C carriers. Findings were assessed in an independent retrospective cohort. Sanger, exome, and short-read and long-read whole-genome sequencing were used for genotyping.

**Results:**

The prospective cohort included 61 carriers: 21 had severe dystonia and 28 had no or minimal dystonia, six of whom carried c.646G>C. Among the 43 participants in the primary analysis, haplotype A was identified in 33 (77%) and haplotype B in eight (19%). All haplotype B carriers had no or minimal dystonia (p=0.003). Haplotype B remained associated with a milder phenotype after including 12 participants with intermediate severity (p=0.006). The retrospective cohort included 147 carriers, 16 of whom carried c.646G>C. After their exclusion, dystonia manifested in 12 (38%) of 32 participants with haplotype B and 63 (64%) of 99 without it (p=0.009). Haplotype B was also associated with lower severity scores (p=0.011). Together, c.646G>C and local haplotype status predicted the phenotype of more than two-thirds of carriers. In silico analyses linked haplotype B to lower *TOR1A* and higher *TOR1B* expression in dystonia-relevant brain regions.

**Conclusion:**

A common local haplotype *in trans* to the disease-causing *TOR1A* variant was strongly associated with reduced penetrance and severity. These findings establish DYT- *TOR1A* dystonia as a model for investigating how local genetic variation contributes to incomplete penetrance in autosomal dominant neurological disorders.

## Full Text Availability

The license terms selected by the author(s) for this preprint version do not permit archiving in PMC. The full text is available from the preprint server.

